# Evaluating the bromodomain protein BRD1 as a therapeutic target in rheumatoid arthritis

**DOI:** 10.1038/s41598-018-29127-w

**Published:** 2018-07-24

**Authors:** Kerstin Klein, Masaru Kato, Mojca Frank-Bertoncelj, Christoph Kolling, Adrian Ciurea, Steffen Gay, Caroline Ospelt

**Affiliations:** 1 Center of Experimental Rheumatology, Department of Rheumatology, University Hospital Zurich, Zurich, Switzerland; 2 Schulthess Clinic, Zurich, Switzerland

## Abstract

Targeting epigenetic reader proteins by small molecule inhibitors represents a new therapeutic concept in autoimmune diseases such as rheumatoid arthritis (RA). Although inhibitors targeting bromodomain protein 1 (BRD1) are in development, the function of BRD1 has hardly been studied. We investigated the therapeutic potential of BRD1 inhibition in joint-resident cells in RA, synovial fibroblasts (SF) and macrophages. The proliferation of SF was decreased upon BRD1 silencing, accompanied by the downregulation of genes involved in cell cycle regulation. Silencing of BRD1 in SF decreased the basal expression of MMP1 but increased TNF-α- and LPS-induced levels of MMP3, IL6 and IL8. In monocyte-derived macrophages (MDM), silencing of BRD1 decreased the LPS-induced expression of TNF-α, but did not significantly affect basal and the TNF-α- and LPS-induced expression of IL6 and IL8. Our data point to a cell type- and a stimulus-specific function of BRD1. Inhibiting BRD1 could have potential beneficial effects in RA via decreasing the proliferation of SF. Anti-inflammatory effects were limited and only observed in MDM.

## Introduction

Histone lysine acetylation, in combination with other post-translational modifications and DNA methylation determines the epigenetic code that regulates gene expression. Enzymes that read and erase histone acetylation marks are involved in regulating pathogenic pathways in rheumatoid arthritis (RA), including inflammation^1^. Bromodomain proteins (BRD) are epigenetic readers of acetylated histones. Targeting BRDs by small-molecule inhibitors has emerged as a new potential therapy in inflammation and arthritis^2^. Inhibitors against the family of bromodomain and extra-terminal (BET) proteins exhibit anti-inflammatory properties^3^ and show beneficial effects in RA synovial fibroblasts (RASF)^4^ and experimental arthritis^5^. First inhibitors targeting members of a distinct BRD family, the bromodomain and plant homeodomain finger-containing (BRPF) family, including BRPF1, BRD1 (BRPF2), and BRPF3 have recently been developed^6,7^, however, they have not been tested regarding their anti-inflammatory potential.

BRD1 has been identified as a subunit of the MOZ/MORF histone acetyltransferase (HAT) complex^8^. BRD1 has been functionally linked to the acetylation of histone 3 lysine 14 (H3K14ac)^9^, a histone mark that co-occurs together with H3K9ac marks at active promoters. Furthermore, H3K14ac marks prime inactive genes for stimuli-dependent activation in mouse embryonic stem cells^10^. In anticitrullinated peptide antibody-positive RA patients, an intronic single nucleotide polymorphism (SNP; rs138845) in *BRD1* was associated with the progression of joint destruction in stage I of a genome-wide association study^11^, providing a potential link between RA and BRD1. In this work, we evaluated the function of BRD1 in joint resident cells, specifically synovial fibroblasts (SF) and macrophages, and explored the potential of BRD1 inhibition as a treatment strategy in RA.

## Results

### Expression of BRD1 in the synovium

**Figure 1:**
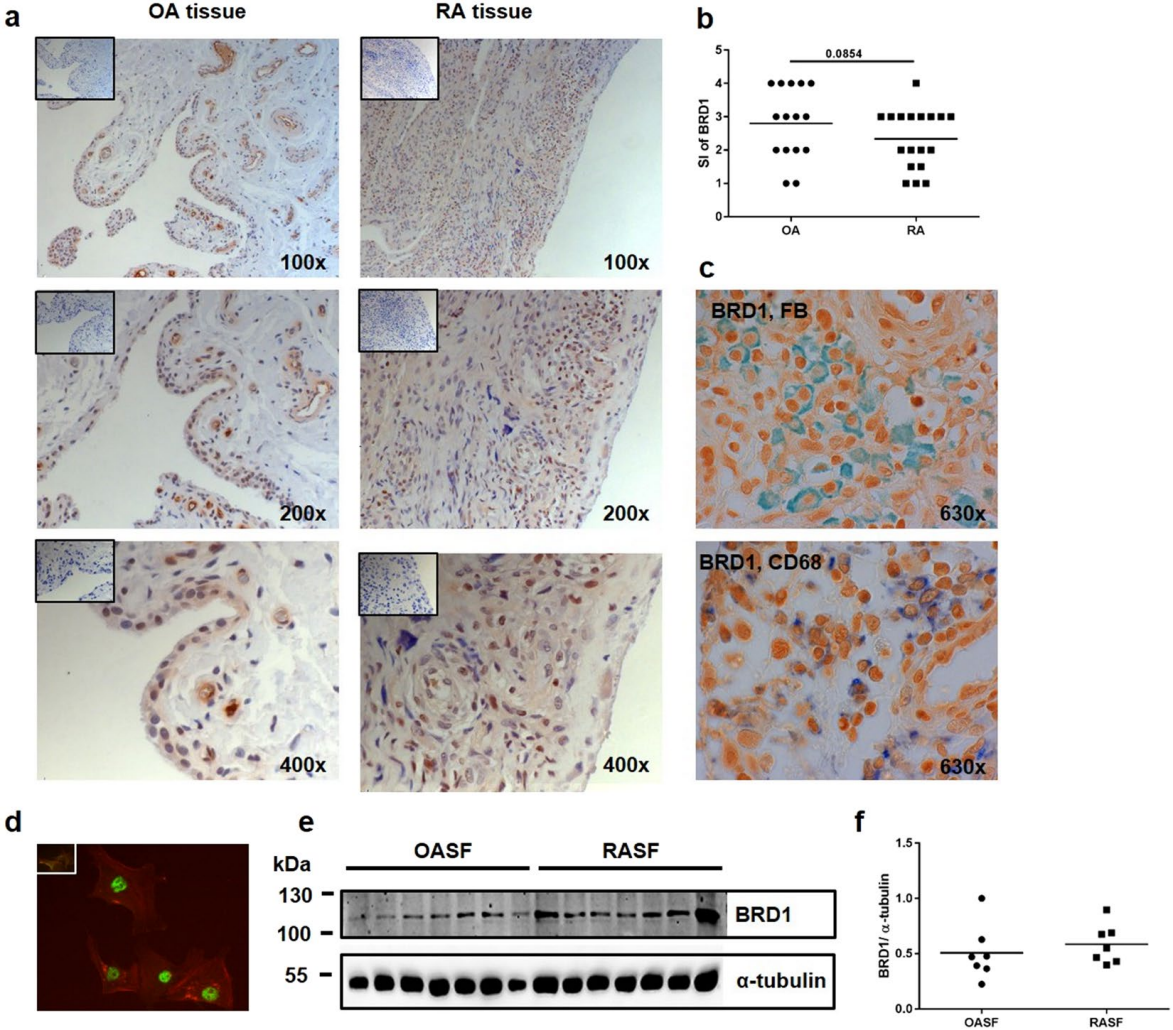
Synovial expression of BRD1. (**a**) Synovial tissues from OA and RA patients were stained with anti-BRD1 (brown) antibodies or isotype control (box). Nuclei were counter stained with hemalaun (blue). Representative pictures are shown from one RA patient (elbow) and one OA patient (shoulder); please see also Supplementary Fig. S1. (**b**) Staining intensities (SI) in OA (n = 15) and RA (n = 18) tissues were scored (1: little staining – 4: strong staining) and difference was analysed by t-test. (**c**) Synovial tissues were double stained with anti-BRD1 (brown) antibodies and anti-CD68 antibodies (blue) or antibodies against prolyl 4-hydroxylase beta, a fibroblast marker (FB, green). Representative pictures are shown from one RA patient (elbow). (**d**) Cultured RASF were stained with anti-BRD1 (green) antibodies or isotype control (box). Actin fibres were stained with phalloidin (red). (**e**) The protein expression of BRD1 in OASF (n = 7) and RASF (n = 7) was analysed by Western blotting. The expression of α-tubulin was used as an endogenous control. Full-length blots are presented in Supplementary Fig. S2. (**f**) Band intensities of Western blots were analysed by densitometry.

BRD1 was comparably expressed in synovial tissues of OA and RA patients (Figs 1a,b and S1), with some heterogeneity between patients. These differences were not due to a different joint of origin^12^ of these tissues (Supplementary Fig. S1). There was no difference in staining intensities of BRD1 between lining and sublining layers of synovial tissues. BRD1 was present in both SF and macrophages (Fig. 1c) as detected by double staining of synovial tissues with antibodies against BRD1 and prolyl 4-hydroxylase beta, a marker for fibroblasts, or CD68, a marker for macrophages. Immunofluorescence microscopy affirmed the nuclear expression of BRD1 in cultured RASF (Fig. 1d). There was no difference in the protein expression of BRD1 in RASF and OASF (Fig. 1e,f), which is in concordance with the results of the tissue staining.

### Silencing of BRD1 reduces proliferation in RASF

**Figure 2:**
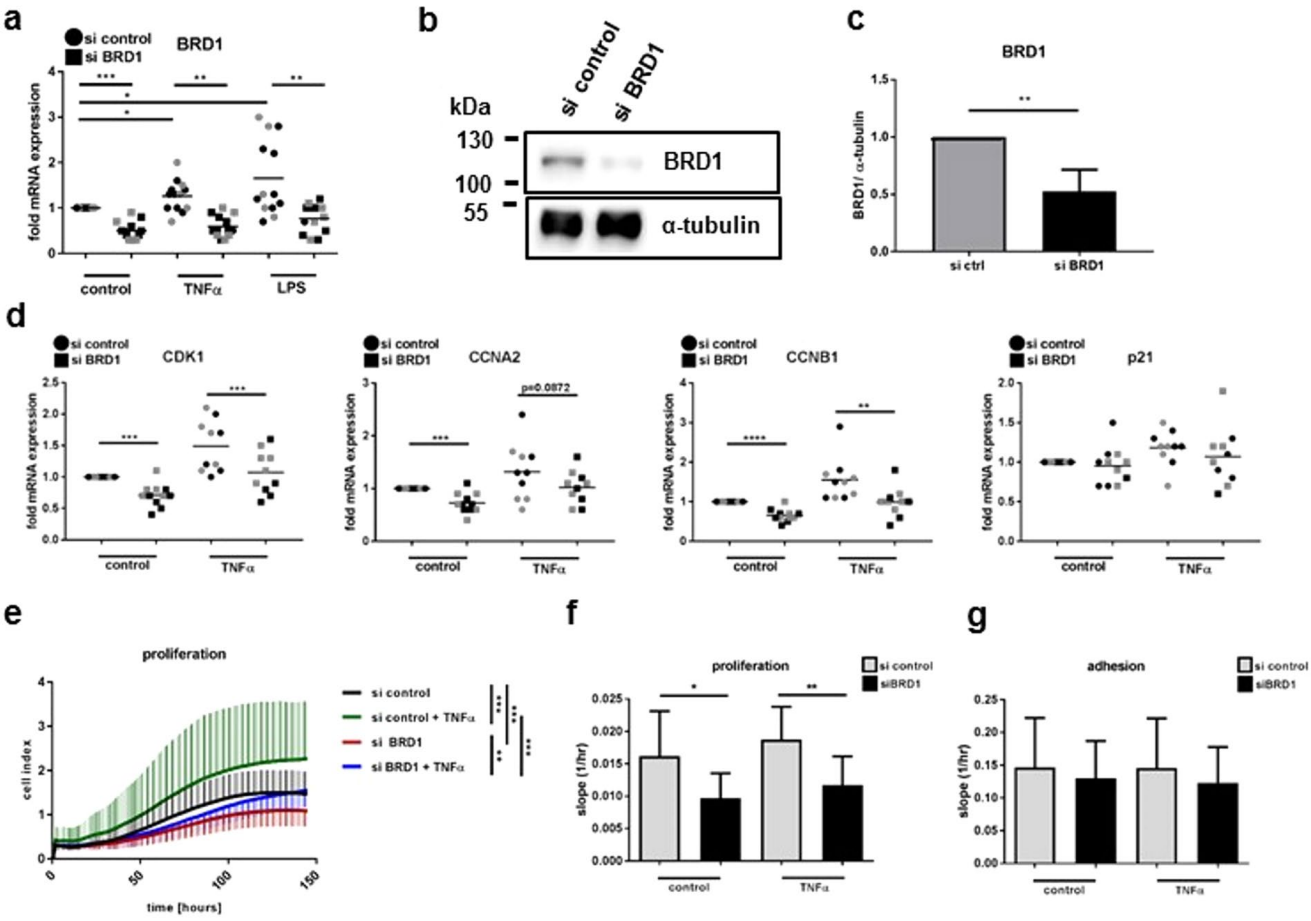
Silencing of BRD1 reduces the proliferation of RASF. RASF and OASF were transfected with siRNAs targeting BRD1 or scrambled siRNAs. 24 h after transfection, cells were stimulated with TNF-α (10 ng/ml) or LPS (100 ng/ml). (**a**) The mRNA and (**b**) protein expression of BRD1 was analysed by quantitative Real-time PCR and by Western blotting. Full-length blots are presented in Supplementary Fig. S3. (**c**) Band intensities of Western blots were analysed by densitometry. (**d**) The mRNA expression of genes involved in cell cycle regulation was analysed in transfected RASF (black; n = 6) and OASF (grey; n = 5) by quantitative Real-time PCR. Differences between groups were analysed by t-test. The proliferation of transfected RASF (n = 6) was analysed in quadruplicates using the Real-time cell-based adhesion and proliferation assays (xCELLigence System) (**e**) during the entire experiment (0–146 hours) and (**f**) during the exponential phase of the curve (40–80 hours). Differences in proliferation were analysed by one-way ANOVA (**e**) or by t-test (**f**). (**g**) The adhesion to plastic was monitored within the first 90 minutes after cell seeding. *p < 0.05, **p < 0.01, ***p < 0.001.

To study its function, we silenced BRD1 in SF in presence and absence of TNF-α and LPS. The pro-inflammatory stimuli TNF-α and LPS increased the expression of BRD1 mRNA (Fig. 2a). The expression of BRD1 was significantly reduced in SF transfected with BRD1 siRNA compared to scrambled transfected SF in unstimulated and stimulated conditions. Silencing of BRD1 was confirmed on the protein level (Fig. 2b,c).

In HeLa cells, p21, a potent inhibitor of cell cycle dependent kinases, has been described as a BRD1 target^13^. In SF, silencing of BRD1 reduced the basal and TNF-α-induced mRNA expression of cyclin dependent kinase (CDK1), cyclin A (CCNA2) and cyclin B1 (CCNB1). The mRNA expression of p21 was not affected by BRD1 silencing (Fig. 2d). To test whether the differences in the gene expression of these cell cycle regulators translate into altered proliferation, we analysed the effect of BRD1 silencing on proliferation of RASF in presence and absence of TNF-α. Silencing of BRD1 decreased the proliferation of RASF, both in presence and absence of TNF-α (Fig. 2e,f). This effect was present during the entire time of the experiment (0–146 hours) (Fig. 2e), as well as during the exponential phase of the proliferation curve (40–80 hours) (Fig. 2f). TNF-α increased the proliferation of scrambled- and siBRD1-transfected cells. However, whereas in scrambled-transfected cells, TNF-α increased proliferation throughout the time of the experiment (0–146 hours), TNF-α increased proliferation only at later stages (70–146 hours) in BRD1 silenced RASF (Fig. 2e). To exclude that the effects on proliferation resulted from an altered adherence of the cells to plastic after silencing of BRD1, we analysed the cell index within the first 90 minutes of the experiment. Silencing of BRD1 in presence and absence of TNF-α had no effect on the ability of RASF to adhere to plastic (Fig. 2g). Furthermore, apoptosis was not changed between scrambled- and BRD1-transfected RASF (Supplementary Fig. S4), excluding that differences in cell death are the cause of the observed changes in proliferation.

### Cell-type specific regulation of gene expression upon silencing of BRD1

**Figure 3:**
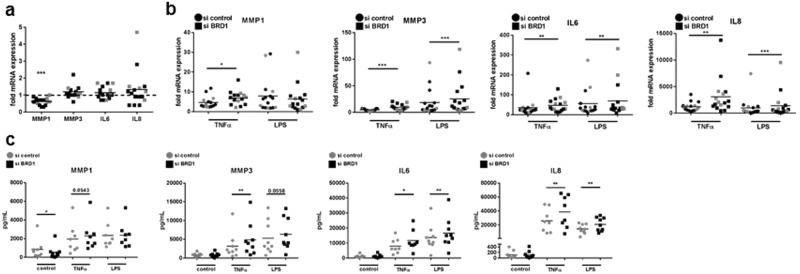
BRD1 regulates the MMP and cytokine production in RASF and OASF. RASF and OASF were transfected with siRNAs targeting BRD1 or scrambled siRNAs. 24 h after transfection, cells were stimulated with TNF-α (10 ng/µl) or LPS (100 ng/µl). The mRNA expression of MMP1, MMP3, IL6, IL8 after silencing in (**a**,**b**) RASF (black; n = 11) and OASF (grey; n = 5) was analysed by quantitative Real-time PCR. (**c**) Effects of BRD1 silencing on MMP1, MMP3, IL6 and IL8 protein secretion in RASF (n = 8–9) was analysed by ELISA. Differences between groups were analysed by t-test. *p < 0.05, **p < 0.01, ***p < 0.001.

Since the inhibition of BET proteins reduced the expression of inflammatory cytokines and MMPs in both RASF^4^ and macrophages^3^, we analysed changes in gene expression in RASF, OASF and monocyte-derived macrophages (MDM) after silencing of BRD1. Silencing of BRD1 in RASF and OASF reduced the basal MMP1 mRNA expression and secretion (Fig. 3a,c) but had no effect on the TNF-α- or LPS-induced expression and secretion of MMP1. In contrast, the TNF-α- and LPS-induced expression and secretion of MMP3, IL6 and IL8 were increased by BRD1 silencing (Fig. 3b,c), without affecting the basal mRNA expression and protein secretion (Fig. 3a,c). These data point to a rather pro-inflammatory effect of BRD1 silencing in SF.

**Figure 4:**
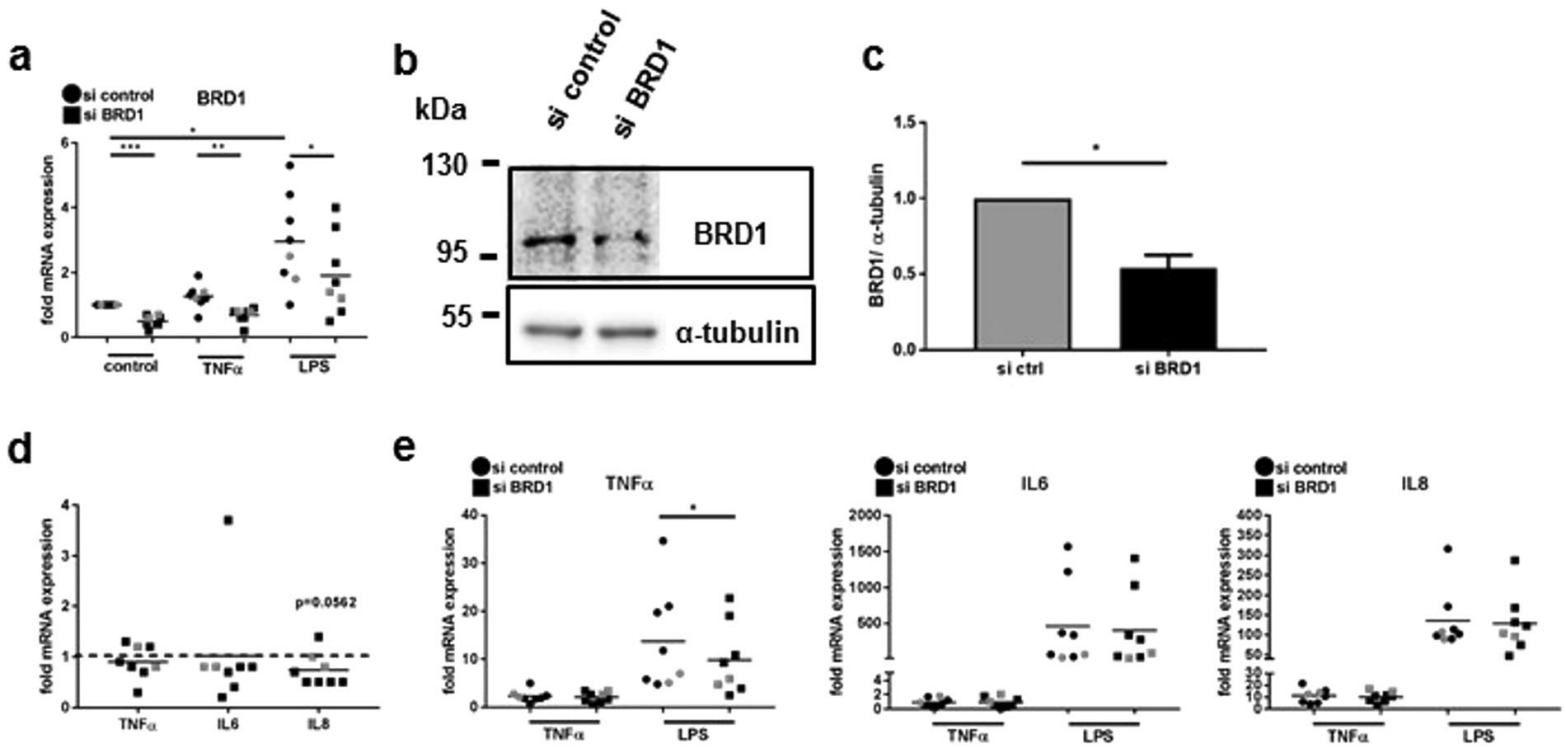
BRD1 regulates the MMP and cytokine production in MDM. MDM were transfected with siRNAs targeting BRD1 or scrambled siRNAs. 24 h after transfection, cells were stimulated with TNF-α (10 ng/µl) or LPS (100 ng/µl). (**a**) The mRNA and (**b**) protein expression of BRD1 was analysed by quantitative Real-time PCR and by Western blotting. Full-length blots are presented in Supplementary Fig. S5. (**c**) Band intensities of Western blots were analysed by densitometry. (**d**,**e**) The mRNA expression of TNFα, IL6, and IL8 after BRD1 silencing in MDM from healthy donors (black; n = 6) and RA patients (grey; n = 2) was analysed by quantitative Real-time PCR. Differences between groups were analysed by t-test. (D) *p < 0.05, **p < 0.01, ***p < 0.001.

Silencing of BRD1 in MDM was confirmed by Real-time PCR (Fig. 3a) and Western blotting (Fig. 3b,c). LPS increased the mRNA expression of BRD1. However, in MDM transfected with BRD1 siRNA, levels were significantly decreased compared to scrambled transfected SF in all conditions (Fig. 4a). Silencing of BRD1 in MDM from healthy donors and RA patients exhibited mild anti-inflammatory effects. The basal expression of IL8 mRNA was slightly but not significantly reduced by silencing of BRD1 in MDM (Fig. 4d, n = 8, p = 0.0562). Furthermore, silencing of BRD1 reduced the LPS-induced expression of TNF-α mRNA (Fig. 4e) but not the TNF-α-induced expression of TNF-α mRNA. This points to a stimulus-dependent regulation of TNF-α in BRD1-silenced MDM. The TNF-α-and LPS-induced expression of IL6 and IL8 were not affected by BRD1 silencing in MDM (Fig. 4e).

## Discussion

Inhibitors targeting the bromodomain of BRD1 (BRPF2) have recently been developed^6,7^ but there is a lack of knowledge on the functional role of BRD1 in adult tissues. In our study, we assessed the role of BRD1 in joint resident cells, SF and macrophages which both contribute to hyperplasia of the synovium and promotion of inflammation found in RA^14^. By conducting silencing experiments in SF and MDM, we analysed here for the first time the functional role of BRD1 regarding its potential role in inflammation, matrix degradation and proliferation. Our silencing experiments suggest a role of BRD1 in the regulation of proliferation of SF. This is in line with silencing of BRD1 in HeLa cells that increased expression of p21, a negative regulator of cell cycle progression^13^. In SF, silencing of BRD1 did not affect the expression of p21 but reduced the expression of CDK1, cyclin A2 and cyclin B2. This suggests that BRD1 regulates the transition of G1/S as well as G2/M phases of the cell cycle in SF. In a panel of cancer cell lines, both BRD1-specific and BRPF pan-inhibitors exhibited a modest or no effects on proliferation^6,7^. Collectively, these differences might reflect cell type-specific effects or point to functional differences between silencing of a multi-domain protein compared to blocking its bromodomain only.

Beside the anti-proliferative effect in SF, we showed that BRD1 regulates cytokine and MMP gene expression in a cell type- and stimulus-specific manner. Effects of BRD1 inhibition on other arthritis-relevant cell types in addition to SF and MDM are likely since BRD1 is expressed also in T- and B-cells^15,16^. BRD1 plays an important role in T cell development and is indispensable for the activation of the *CD8* gene during early thymocyte development^15^. In SF, silencing of BRD1 exhibited rather pro-inflammatory and pro-destructive functions in presence of inflammatory stimuli. We did not observe any differences in SF from RA and OA patients, in accordance with the comparable expression of BRD1 in RA and OA tissues. Furthermore, there were no differences after BRD1 silencing in SF derived from different joint origin with respect to measured gene products. In MDM the LPS- induced expression of TNF-α was decreased by BRD1 silencing. This anti-inflammatory effect in MDM was stimulus-specific, since TNF-α did not affect the expression of TNF-α mRNA upon BRD1 silencing. This is in line with previous studies that showed a stimulus-specific expression of TNF-α in different cell types *in vitro* and *in vivo*^17–19^. A cell-type specific regulation of IL6 in SF and CD14^+^ monocytes has been described also previously by *Noss et al*. who identified a single nucleotide polymorphism in the *IL6* promoter that affected only the expression of IL6 in SF from RA and OA patients but not in monocytes^20^. We have recently showed a cell-type specific regulation of a larger panel of cytokines, chemokines and MMPs in SF and MDM upon repeated stimulation with LPS. This coincided with a different epigenetic architecture at the promoters of these genes in unstimulated SF and monocytes, leading to a cell-type specific regulation of these genes after stimulation with LPS^21^. BRD1 is associated with a HAT complex adding H3K14ac marks to active gene promoters^9^. H3K14ac marks are rapidly induced in promoter regions of *IL6*, *IL8* and *TNF*-*α* by inflammatory stimuli in different cell types^22,23^. In THP-1 monocytes, H3K14ac marks preceded the recruitment of the p65 in the TNF-α promoter upon stimulation with S100b proteins^22^, suggesting a role of H3K14ac in the activation of the TNF-α promoter. Unfortunately, there are no public data sets on H3K14ac marks available that would enable a comparison of this histone modification in inflammatory gene promoters in macrophages and SF or other fibroblasts.

In IL-4-stimulated macrophages, pan-BRPF inhibitors modulated the secretion of CCL-22. However, depending on the concentration of these inhibitors, both a decreased and an increased secretion of CCL-22 were observed^7^, making the interpretation of the results difficult. In addition, BRPF inhibitors were mentioned to impair the RANKL-induced differentiation of monocytes into bone resorbing osteoclasts^7^. These results, together with the reported *BRD1* SNP (rs138845) and our findings suggest a potential role of BRD1 in the pathogenesis of arthritis. In contrast to BET protein inhibition, that has a broad range of anti-inflammatory and anti-proliferative effects in diverse cell types *in vitro* and *in vivo*^24^, BRD1 inhibition rather exhibits a cell type and stimulus-specific functions with only limited anti-inflammatory effects with respects to the studied gene products and cell types. Therefore, BRPF inhibitors should be tested in many different cell types and under different stimulatory conditions.

## Methods

### Patient samples and cell preparation

Synovial fibroblasts of RA and osteoarthritis (OA) patients and monocyte-derived marcophages (MDM) from healthy volunteers and RA patients were cultured as described^21^. All patients fulfilled the criteria for the classification of RA^25^ and OA^26^. The characteristics of patients undergoing joint replacement surgery is shown in Table 1. The studies were approved by the local ethic committee of the University Hospital Zurich, Switzerland. Informed consent was obtained from all patients and healthy volunteers. All experiments have been performed in accordance with the institutional guidelines.

**Table 1:** Characteristics of patients.

	RA (n = 27)	OA (n = 9)
age at time of joint replacement (years, mean (range))	65 (36–82)	71 (58–83)
sex (female/male)	26/1	14/2
disease duration (years, mean (range))	23 (2–43); ND 2	ND
RF (positive/negative)	23; ND 4	ND
smoker/non-smoker	3/24	1/15; ND 1
CRP (mean ± SD)	21.8 ± 22.4; ND 4	2.8 ± 3.9; ND 4
medication	ND 5	ND 17
DMARDs	15	
NSAIDs	5	1
steroids	8	
biologics	9	
joints		
hand	5	0
ankle	1	0
shoulder	8	2
elbow	8	0
knee	4	13
hip	1	3

*CRP* C reactive protein, *DMARDs* disease modifying antirheumatic drugs, *ND* not determined, *NSAIDs* nonsteroidal anti-inflammatory drugs, *SD* standard deviation.

### Transfection experiments

3.5 × 10^5^ RASF or OASF and 6.5 × 10^5^ MDM were transfected with a pool of two different siRNAs (2 × 0.75 μM) targeting BRD1 or scrambled siRNAs (Qiagen) as a control using the Amaxa Basic Nucleofector Kit for Primary Mammalian Fibroblasts or the Human macrophage nucelofector kit (Lonza). 24 h after transfection cells were stimulated with TNF-α (10 ng/ml) or LPS (100 ng/ml) as indicated for another 24 h.

### Immunohistochemistry and Immunofluorescence

After deparaffinization, tissue sections of RA and OA patients were pre-treated with citrate buffer (10 mM sodium citrate, pH 6.0). Endogenous peroxidase activity was disrupted with 3% H_2_O_2_. Slides were permeabilized with 0.1% Triton in PBS. Nonspecific protein binding was blocked with 5% goat serum in antibody diluent (Dako). Polyclonal rabbit anti-BRD1 antibodies (abcam) and rabbit IgG1 were applied over night at 4 °C. Slides were washed in PBS-T (0.05% Tween 20 in PBS) and incubated with biotinylated goat anti-rabbit antibodies (Jackson ImmunoResearch). The signal was amplified with ABC reagent for peroxidase (Vector Laboratories, Burlingame, CA) and detected with 3,3′-diaminobenzidine (Vector Laboratories). Staining intensities (SI) of tissues were scored (1: little staining – 4: strong staining) independently by two researches. For double staining, slides were stained with either the fibroblast marker prolyl 4-hydroxylase beta (Acris), CD68 (DakoCytomation) or IgG the day after staining for BRD1. Slides were washed in PBS-T and incubated with biotinylated goat anti-mouse antibodies. The signal was amplified with ABC reagent and detected with either Vector blue (Vector laboratories) or HistoGreen (Linaris). For immunofluorescence stainings, RASF were cultured in chamber slides (Lab-Tek, Nunc), fixed with 4% paraformaldehyde and permeabilized with tris-buffered saline (TBS) containing 0.1% Triton X-100. Nonspecific binding was blocked with 5% goat serum. Slides were incubated with polyclonal rabbit anti-BRD1 antibodies and rabbit IgG1 overnight at 4 °C. After washing, slides were incubated with FITC- conjugated secondary antibodies (Thermo Scientific). Actin filaments were stained with phalloidin (Sigma-Aldrich). Slides were covered with fluorescent mounting medium (Dako) and analysed with a fluorescence microscope (Axio Imager).

### Quantitative Real-time polymerase chain reaction (RT-PCR)

Total RNA was isolated from cells using the ReliaPrep RNA Cell Miniprep System (Promega) including on-column DNaseI digest and reversed transcribed. Real-time PCR was performed using SYBR green (Life Technologies). Primer sequences are available on request. Constitutively expressed human ribosomal protein large P0 (RPLPO) was measured for internal standard sample normalization. Relative mRNA expression was calculated by the comparative threshold cycle method (ΔΔCt).

### Western blotting

Western blotting was performed as described in detail previously^4^. Membranes were probed with antibodies against BRD1 (abcam) or α-tubulin (abcam). As secondary antibodies, horseradish peroxidase-conjugated goat anti-rabbit or goat anti-mouse antibodies (Jackson ImmunoResearch) were used. Signals were detected using the ECL Western blotting detection reagents (GE Healthcare) and the Alpha Imager Software system (Alpha Innotech).

### ELISA

MMP1, MMP3, IL6 and IL8 were measured using the human total MMP-1 ELISA kit, the human total MMP-3 ELISA kit, the human IL-6 ELISA Set, and the human IL-8 ELISA Set (BD Biosciences), respectively in cell culture supernatants.

### Adhesion and proliferation assay

Proliferation and adhesion (towards plastic) of transfected RASF was analysed using an impedance-based system for Real-time proliferation and adhesion assays (xCELLigence System, Bucher Biotec AG). Cells were seeded into E-plates 24 h after transfection at a densitiy of 2500 cells/well. TNF-α (10 ng/ml) was added simultaneously during seeding. Each condition was analysed in quadruplicates. Impedance changes were recorded every 15 minutes (0–24 hours) and every 30 minutes (24–146 hours). Adhesion was analysed over the first 90 minutes of the experiment. Proliferation was analysed during the exponential phase of the slopes (40–80 hours) and over the entire period of the experiment (0–146 hours).

### Analysis of cell death

SF were incubated with FITC Annexin V (BD Biosciences) and propidium iodide (Sigma-Aldrich) for 15 minutes at room temperature in the dark, as recommended by manufacturer’s protocol (BD Biosciences), and analysed by flow cytometry (FACSCalibur, BD Biosciences).

### Statistical analysis

Statistical analysis on data sets was carried out by using the GraphPad Prism program (GraphPad Software, San Diego, CA). N numbers in all experiments represent biological samples from different patients. Differences between experimental groups were analyzed by paired t-test or Wilcoxon signed rank test for analysis of basal expression. Differences in proliferation (0–146 h) were analysed by one-way ANOVA. Data are reported as means ± standard deviations. P values < 0.05 were considered significant. Raw data files are available on request.

## Electronic supplementary material

Dataset 1
