# Supplementary material for: Evaluating the bromodomain protein BRD1 as a therapeutic target in rheumatoid arthritis
Source: Sci Rep. 2018 Jul 24;8:11125. doi: 10.1038/s41598-018-29127-w (PMC6057939; doi:10.1038/s41598-018-29127-w)
Supplement: Supplementary file 1 — Dataset 1 [file 41598_2018_29127_MOESM1_ESM.pdf]

Evaluating the bromodomain protein BRD1 as a therapeutic target in rheumatoid arthritis

Kerstin Klein[1*], Masaru Kato[1], Mojca Frank-Bertoncelj[1], Christoph Kolling[2], Adrian Ciurea[1], Steffen Gay[1], Caroline Ospelt[1]

Supplementary information

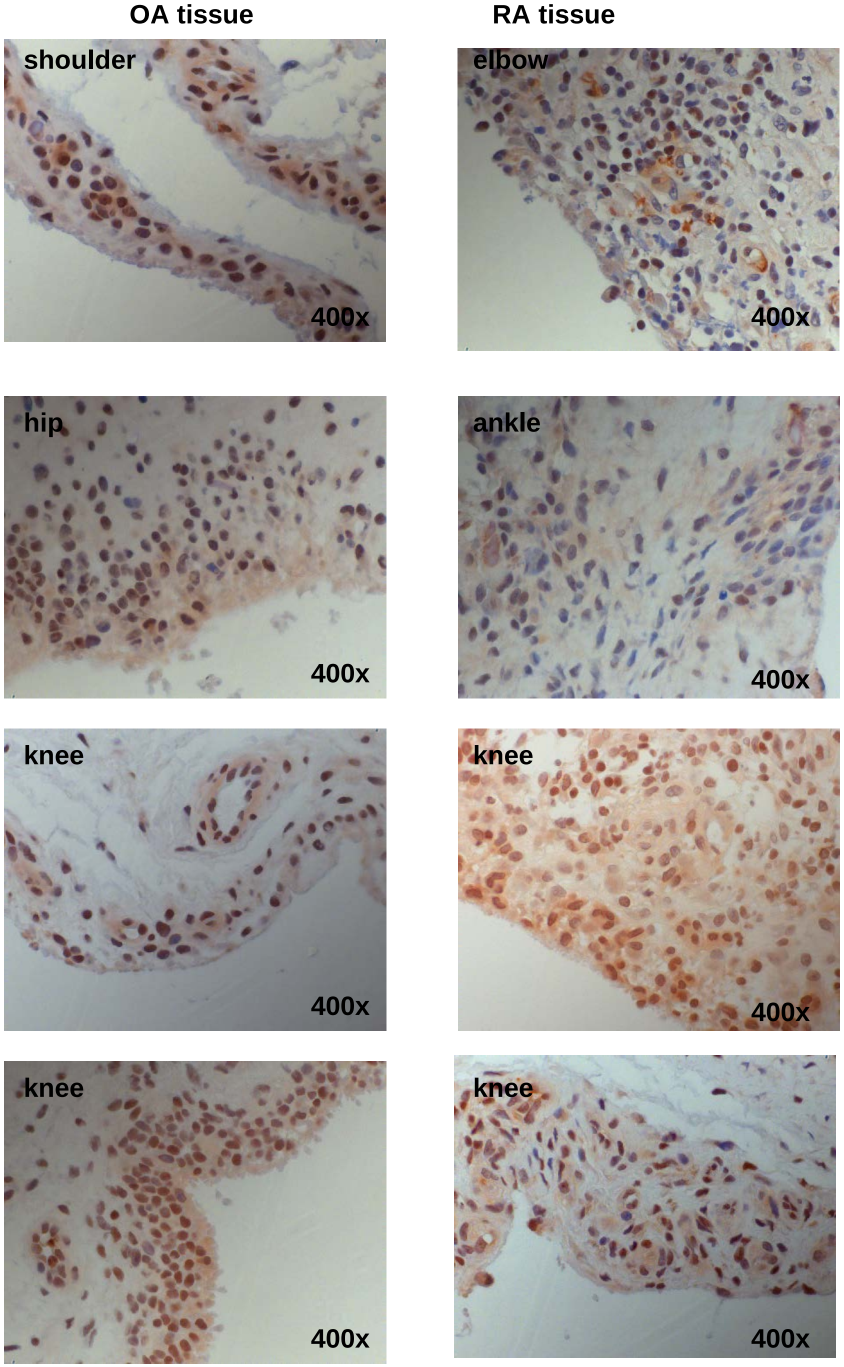

Supplementary Figure S1. Synovial tissues from OA and RA patients were stained with anti-BRD1 (brown) Nuclei were counter stained with hemalaun (blue). The joint of origin is mentioned for each picture. Representative pictures are shown in figure 1.

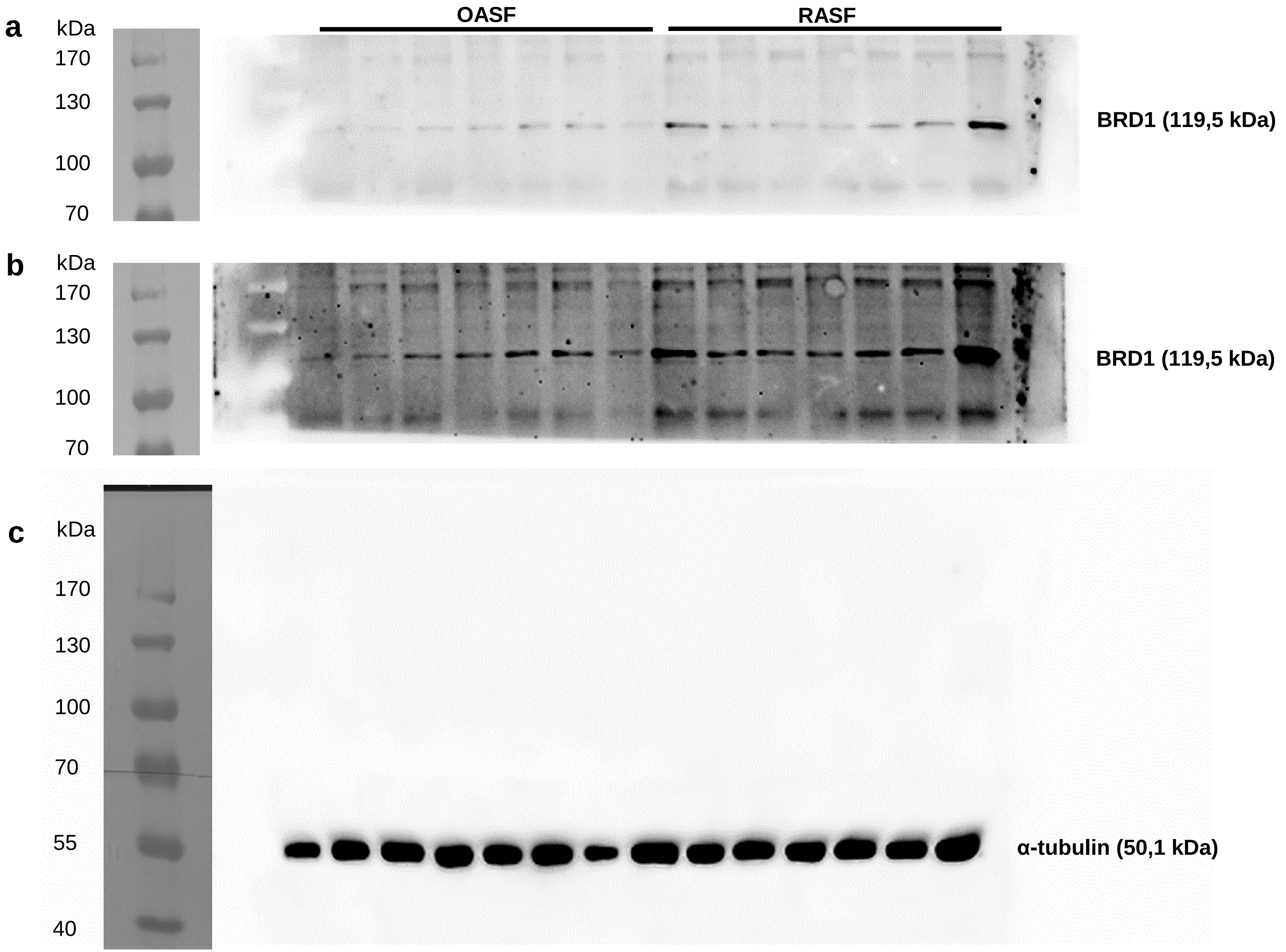

Supplementary Figure S2. Full-length blots corresponding to Figure 1. Size markers are presented on the left side of corresponding blots. The protein expression of BRD1 in osteoarthritis (OASF; n=7) and rheumatoid arthritis synovial fibroblasts (RASF; n=7) was analysed by Western blotting. (a) Short and (b) long exposures of BRD1. (c) The expression of α-tubulin was used as an endogenous control. Expected protein sizes are shown in parentheses.

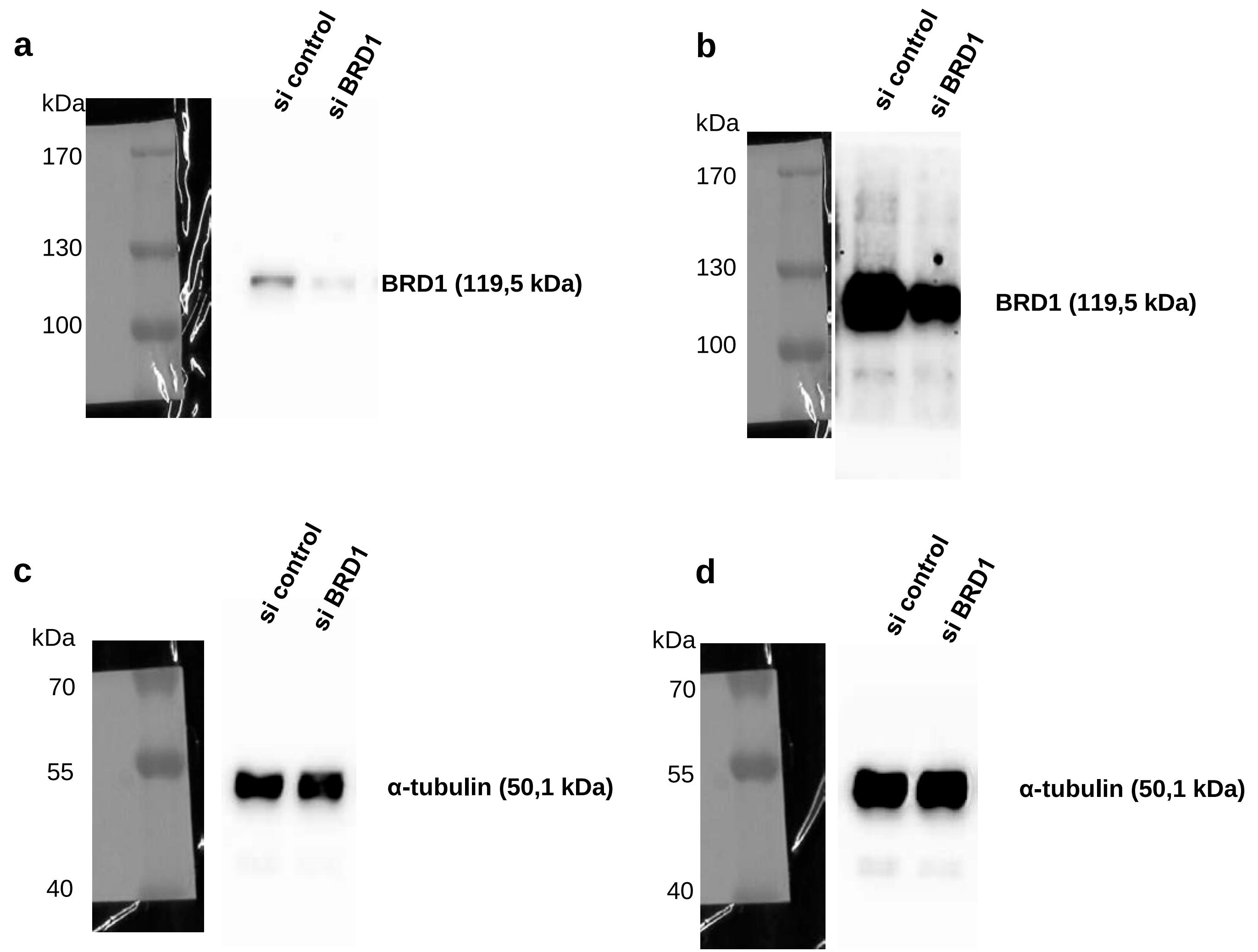

Supplementary Figure S3. Full-length blots corresponding to Figure 2. Size markers are presented on the left side of corresponding blots. Rheumatoid arthritis synovial fibroblasts (RASF) were transfected with siRNAs targeting BRD1 or scrambled siRNAs. The protein expression of BRD1 was analysed by Western blotting. (a) Short and (b) long exposures of BRD1. (c) Short and (d) long exposures of α-tubulin which was used as an endogenous control. Expected protein sizes are shown in parentheses.

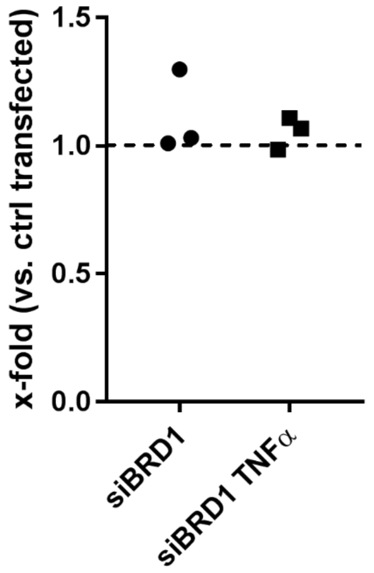

Supplementary Figure S4. RASF (n=3) were transfected with siRNAs targeting BRD1 or scrambled siRNAs. 24h after transfection, cells were stimulated with TNF-α (10 ng/µl). Dead cells were evaluated by Annexin V/ PI staining followed by FACS analysis.

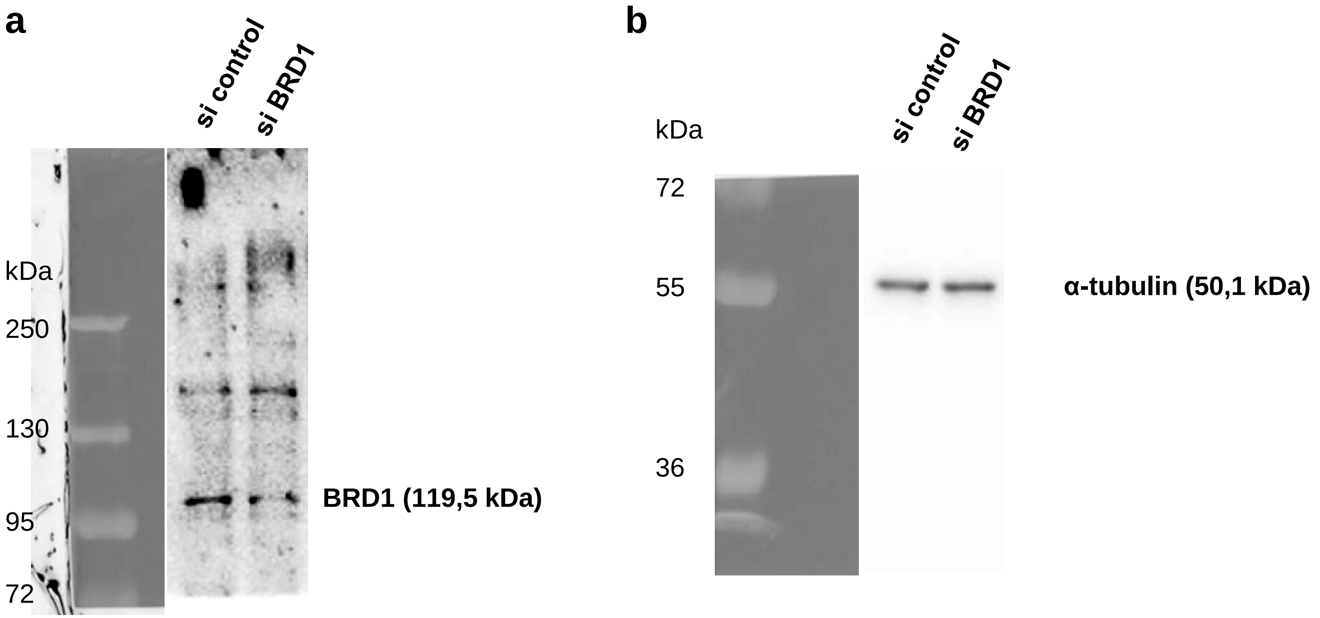

Supplementary Figure S5. Full-length blots corresponding to Figure 4. Size markers are presented on the left side of corresponding blots. Monocyte-derived macrophages (MDM) were transfected with siRNAs targeting BRD1 or scrambled siRNAs. (a) The protein expression of BRD1 was analysed by Western blotting. (b) The expression of α-tubulin was used as an endogenous control. Expected protein sizes are shown in parentheses.
